# Relationship between multimorbidity treatment burden and chronic disease self-efficacy in the elderly population of China

**DOI:** 10.3389/fmed.2025.1521189

**Published:** 2025-04-03

**Authors:** Yiwei Guo, Jialong Tan, Weigui Shi, Jing Bai, Jian Wang

**Affiliations:** ^1^Dong Fureng Economic and Social Development School, Wuhan University, Wuhan, Hubei, China; ^2^Shanxian Central Hospital, Heze, Shandong, China; ^3^Center for Health Economics and Management at the School of Economics and Management, Wuhan University, Wuhan, Hubei, China

**Keywords:** multimorbidity, chronic diseases, elderly population, treatment burden, chronic disease self-efficacy

## Abstract

**Objectives:**

This study aimed to evaluate the risk factors associated with the treatment burden of multimorbidity among older Chinese adults and to investigate the relationship between treatment burden and chronic disease self-efficacy.

**Methods:**

Data for this study were collected from a population-based investigation of individuals aged 60 years and older with at least two chronic diseases, conducted by Wuhan University. A structured questionnaire was employed to collect demographic information, chronic disease status, multimorbidity treatment burden (assessed using the Multimorbidity Treatment Burden Questionnaire, MTBQ), and chronic disease self-efficacy (assessed using the Self-Efficacy for Managing Chronic Disease 6-item scale, SEMCD6). The Pearson chi-square test and logistic regression were used to analyze the data.

**Results:**

A total of 1,599 individuals with a mean age of 71.48 years were enrolled in our study, of whom 13.01% were over 80 years. The proportion of low, medium, and high multimorbidity treatment burden was 30.42, 13.88, and 15.21%, respectively. For elderly individuals; residence; gender; age; high school education experience; comorbidities with stroke, chronic respiratory disease, Parkinson’s disease, dementia, psychological disorders; and individuals who had 6 or more chronic diseases were found to be significantly associated with the MTBQ score. A higher MTBQ score was significantly associated with a lower SEMCD6 score.

**Conclusion:**

The findings suggest that lower socioeconomic status and comorbidities with stroke, cognitive diseases, and mental disorders contribute to a higher perceived MTBQ score. Moreover, a higher level of multimorbidity treatment burden may potentially lead to poorer motivation for health management behaviors.

## Introduction

Complex medical tasks impose a heavy burden on individuals with multiple chronic conditions, especially long-term conditions that persist for years or even a lifetime. The goal of chronic disease management is shifting from merely treating diseases to comprehensive health management. In recent years, a growing consensus has emerged on the importance of engaging individuals with multiple chronic conditions in the healthcare process, emphasizing their active participation as a critical component of effective disease prevention and control strategies (1). Substantial financial and resource investments have been allocated to manage chronic diseases, creating a significant burden on both society and individuals. Meanwhile, patients and caregivers invest time and energy to following treatment regimens, self-monitoring, and health management (2). Accumulating burdens lead to challenges for the healthcare system and individuals due to the increasing burden of long-term, even life-limiting treatment. With the overwhelming tasks imposed on those populations, the healthcare outcome for them is deteriorating.

The treatment burden is exacerbated by complex medical prescriptions, poor communication with healthcare professionals, multiple conditions, lack of motivation, and difficulty in mobilizing resources (3–6). Prolonged illness duration, exacerbated by aging, may lead to cognitive impairment and frailty (7). Furthermore, chronic diseases are often accompanied by psychological issues, which impose an additional burden on disease management tasks (8, 9). Becker (10) developed the Health Belief Model, which posits that self-efficacy is closely linked to patients’ willingness and ability to mobilize resources to achieve their health goals. This relationship has been widely studied (10–12). Moreover, the level of self-efficacy may affect individuals’ behaviors, such as adherence to medical regimes, health behaviors, and health outcomes. Patients with high self-efficacy are better equipped to manage their conditions, adhere to treatment plans, and maintain a higher quality of life (13–18). In the process of regulating chronic disease, failure to complete medical tasks may result in an increased treatment burden and compromised health and wellbeing for patients (14, 19). When evaluating the effects of multimorbidity healthcare outcomes, self-efficacy should be taken into consideration (20).

To understand the concept of treatment burden, several studies have proposed measurement frameworks, particularly in the context of multimorbidity (21, 22). A notable contribution comes from a research team that developed two key measurement tools: the Treatment Burden Questionnaire (TBQ) and the Patient Experience with Treatment Satisfaction Questionnaire (PET). These tools were designed to assess treatment burden from the perspectives of clinical outcomes and patient satisfaction, respectively. Their findings indicated that lower self-efficacy is associated with a perceived lack of confidence among patients in managing their health conditions (19, 23). Subsequently, researchers developed the multimorbidity treatment burden questionnaire (MTBQ), which provides a more comprehensive view of daily health management by focusing on aspects such as care coordination, conflicting treatments, social support, and cumulative burden (24). The Chinese version of MTBQ has been developed and has demonstrated good reliability and validity among elderly populations (25). However, studies focusing on the correlation between multimorbidity treatment burden and chronic disease self-efficacy are rare, highlighting a critical gap in the literature.

Notably, China is home to the largest population of elderly individuals all over the world. The population aged 60 and older accounts for 18.70% of the total population, of which approximately 75% have at least one non-communicable chronic disease and three-quarters of them suffer from two or more chronic diseases (26, 27). In 2017, the Communist Party of China Central Committee and the State Council jointly introduced a medium- and long-term plan for the prevention and control of chronic diseases (28). This plan highlights the importance of reducing morbidity, especially for the burden of priority diseases. Furthermore, it calls for clarity in the responsibilities of government entities, medical and health institutions, families, individuals, and other stakeholders to strengthen health management. Shanxian County is recognized as one of the hometowns of longevity in Shandong Province. The resident population is 1.026 million, of which the proportion of individuals 60 years or above and 65 years or above is 20.53 and 15.89%, respectively. Shanxian County, a city with a long historical background, epitomizes the representation, integrity, and sustainability of human longevity in China (29). Furthermore, Shanxian County connects eight counties across Shandong, Henan, Anhui, and Jiangsu provinces, ensuring the representativeness of the sample for research.

Therefore, the primary objectives of this study are: (1) to investigate the treatment burden of multimorbidity among elderly individuals from the perspectives of medication and treatment, medical-related, and daily self-health management; (2) to identify factors influencing multimorbidity treatment burden; and (3) to explore the relationship between multimorbidity treatment burden and chronic disease self-efficacy. Accordingly, this study will add evidence to current knowledge and provide evidence-based recommendations for health management.

## Methods

### Data collection and inclusion/exclusion criteria

The sample was derived from the “Chinese Elderly Happiness Survey” conducted by Wuhan University Dong Fureng Economic & Social Development School in Shanxian County, Shandong Province. A structured questionnaire was employed to collect demographic information, including the condition of chronic diseases, multimorbidity treatment burden, chronic disease self-efficacy, and psychological status of participants. Participants were randomly selected from 16 communities attached to two districts (2/4, select two out of four districts) and 16 villages attached to four townships (4/18) in April 2023. A total of 3,483 individuals over 60 years of age were recruited in our study. Samples without chronic disease, those with only one chronic disease, or individuals not taking any medications to manage chronic conditions were excluded from the study. Finally, a total of 1,599 participants were enrolled in our study for the purpose of analyzing the relationship between multimorbidity treatment burden and chronic disease self-efficacy.

### Measures

#### Status of chronic diseases

A total of 22 chronic diseases were investigated in this survey, with questions such as “Have you ever had a doctor tell you that you have 01hypertension/ 02diabetes/ 03heart diseases/ 04stroke, CVD/ 05bronchitis, emphysema, pneumonia, asthma/ 06tuberculosis/ 07glaucoma/ 08cataracts/ 09cancer(malignant tumor)/ 10gastric or duodenal ulcer/ 11Parkinson’s disease/ 12bedsores/ 13arthritis/ 14dementia/ 15epilepsy/ 16dyslipidemia/ 17rheumatism or rheumatoid disease/ 18chronic nephritis/ 19mammary hyperplasia/ 20uterine fibroid(female)/ 21prostatic disease(male)/ 22hepatitis?”

It was reported that cardiovascular diseases, cancer, chronic obstructive pulmonary disease, and diabetes are classified as major chronic diseases, accounting for a high death rate in China (30). Consequently, hypertension, diabetes, heart disease, stroke, bronchitis, emphysema, pneumonia, asthma, and cancer were identified as major chronic conditions. Additionally, cognitive diseases such as Parkinson’s disease and dementia, which may impair patients’ memory and comprehension functions, were also analyzed in this study.

#### Self-reported anxiety

The Generalized Anxiety Disorder questionnaire (GAD-7) was developed by Spitzer et al. (31), which serves as a self-rated tool for screening anxiety disorders, particularly in generalized anxiety disorder. The general recommended scale scores are 5 ~ 9 points for mild anxiety, 10 ~ 14 points for moderate anxiety, and ≥ 15 points for severe anxiety. In this study, a total score of more than 5 was defined as anxiety in the elderly, with the variable value set as “1.”

#### Self-reported depression

The Geriatric Depression Scale (GDS-15) was used to assess the mental health status of the elderly (32). It includes factors such as unpleasantness, apathy and anxiety, loss of disappointment, memory loss, and social activity reduction. The 2-level scoring method is adopted. The answer “yes” is 1 point, and the answer “no” is 0 points. There are five reverse scoring items, and the higher the score, the more obvious the depressive symptoms. Referring to the recommended cut-off value, a total score of more than 5 was defined as depression in the elderly, with the variable value set as “1” in this study (33).

#### Multimorbidity treatment burden

The Chinese version of the Multimorbidity Treatment Burden Questionnaire (MTBQ) was adopted to measure the burden of chronic diseases in the elderly (25). This questionnaire has 12 items, reflecting patients’ investment of time and effort to manage their chronic conditions, including three dimensions: medication and treatment, medical-related, and daily self-health management. A Likert 5-level scoring method is adopted; “4″ means “extremely difficult” and “0″ means “not difficult or not apply.” The score of each dimension is the accumulated score of each item, and the total score is calculated by multiplying the average score of 12 items by 25 [( $\sum_{i=1}^{12}item_{i}$ )/12*25]. It is reported as a score ranging from 0 to 100, categorized into four grades: no burden (score 0), low burden (score 1–9), medium burden (10–21), and high burden (≥22).

#### Chronic disease self-efficacy

The Chinese version of the Self-Efficacy for Managing Chronic Disease 6-item scale (SEMCD6), designed by Stanford University in the United States, was adopted to evaluate the elderly’s self-assessment of their chronic disease management capabilities. This questionnaire reflects the elderly’s confidence in chronic disease symptom management, role function, emotional control, communication with healthcare providers, and other aspects (34). The 1–10 score method was adopted. A higher score indicates a higher level of chronic disease self-management efficacy, and an average score of 7 or below was defined as “low level,” with the value set as “1.”

### Statistical analysis

Logistic regression analysis was conducted to examine the risk factors (age and number of chronic diseases) of multimorbidity treatment burden and the association between the MTBQ score and chronic disease self-efficacy, respectively. The Pearson chi-square test was applied to identify the association between demographic variables (gender, residence, and education) and comorbid disease variables with multimorbidity treatment burden. The odds ratio (OR) values and 95% confidence intervals (CIs) were reported for each risk factor. A *p*-value (two-tailed) less than 0.05 was considered statistically significant. All the analyses were performed using SPSS22.0.

## Results

### Characteristics of participants

Demographic characteristics and health-related conditions of participants are presented in Table 1. A total of 868 (275 males and 593 females) and 731 (280 males and 451 females) participants were investigated in urban and rural areas, respectively. The mean age of individuals was 71.48 (range: 60–95), in which 13.01% of the elderly were over 80 years of age and 196 (12.26%) individuals had a high school education or above. The majority of participants only had two or three kinds of chronic diseases (51.53%), and the three most prevalent chronic diseases among the elderly were hypertension (73.17%), heart diseases (50.47%), and stroke (24.58%).

**Table 1:** Characteristics, MTBQ score, MAQ score, and SEMCD6 score of participants.

Variables	*N*^1^	*n* (%)
Residence	1,599	
Urban		868 (54.28)
Rural		731 (45.72)
Sex	1,599	
Male		555 (34.71)
Female		1,044 (65.29)
Age	1,599	
60–69		671 (41.96)
70–79		720 (45.03)
≥80		208 (13.01)
High school degree or above	1,599	
Yes		196 (12.26)
No		1,403 (87.74)
Numbers of chronic diseases	1,599	
2		455 (28.46)
3		369 (23.08)
4		269 (16.82)
5		188 (11.76)
≥6		318 (19.89)
Hypertension	1,599	
Yes		1,170 (73.17)
No		429 (26.83)
Diabetes	1,599	
Yes		370 (23.14)
No		1,229 (76.86)
Heart diseases	1,599	
Yes		807 (50.47)
No		792 (49.53)
Stroke	1,599	
Yes		393 (24.58)
No		1,206 (75.42)
Chronic respiratory disease	1,599	
Yes		356 (22.26)
No		1,243 (77.74)
Cancer	1,599	
Yes		39 (2.44)
No		1,560 (97.56)
Parkinson’s disease	1,599	
Yes		42 (2.63)
No		1,557 (97.37)
Dementia	1,599	
Yes		45 (2.81)
No		1,554 (97.19)
Self-reported depression	1,594	
Yes		528 (33.12)
No		1,066 (66.88)
Self-reported anxiety	1,594	
Yes		568 (35.63)
No		1,026 (64.37)
MTBQ	1,578	
No burden		639 (40.49)
Low burden(1–9)		480 (30.42)
Medium burden(10–22)		219 (13.88)
High burden(≥22)		240 (15.21)
SEMCD6	1,599	
Yes		510 (31.89)
No		1,089 (68.11)

^1N = sample size.^

### Multimorbidity treatment burden score

The proportion of missing values and “does not apply” responses did not exceed 0.7 and 11.60%, respectively, for each question. The top three treatment burdens were paying for prescriptions and over-the-counter medication or equipment, monitoring medical conditions, and seeing many different health professionals according to the mean value (Supplementary Table 1). All the questions were included in the analysis. A total of 459 individuals had an MTBQ score higher than 10, indicating that these individuals experienced low (30.42%), medium (13.88%), and high (15.21%) treatment burdens due to multimorbidity, respectively (Table 1).

### Risk factors related to MTBQ score

The results of the chi-square test and logistic regression of high treatment burden risk are displayed in Table 2. Multimorbidity treatment burden was significantly associated with residence (rural vs. urban, OR = 3.05, 95%CI = 2.35–3.97), gender (female vs. male, OR = 1.39, 95%CI = 1.07–1.81), age (age ≥ 80 vs. 60 ≤ age ≤ 69, OR = 1.62, 95%CI = 1.07–2.43), and high school education experience (no vs. yes, OR = 5.46, 95%CI = 2.46–12.11). Regarding comorbidities with physical chronic conditions, we observed a significant correlation between stroke (OR = 1.610, 95%CI = 1.26–2.05), chronic respiratory disease (OR = 1.33, 95%CI = 1.03–1.72), Parkinson’s disease (OR = 2.45, 95%CI = 1.61–3.74), and dementia (OR = 1.33, 95%CI = 1.86–4.04) and a higher MTBQ score. Individuals with six or more chronic diseases had a higher risk of high MTBQ scores compared to those with two chronic diseases (OR = 2.71, 95%CI = 1.83–4.01).

**Table 2:** Correlation between independent variables and MTB among the elderly people.

Variables	OR	95%CI	*p*
Residence			
Urban	ref		
Rural	3.050	2.35–3.97	<0.001
Sex			
Male	ref		
Female	1.390	1.07–1.81	0.010
Age			
60–69	ref		
70–79	1.200	0.89–1.62	0.230
≥80	1.620	1.07–2.43	0.020
High school degree or above			
Yes	ref		
No	5.460	2.46–12.11	<0.001
Numbers of chronic diseases			
2	ref		
3	1.360	0.9–2.06	0.150
4	1.240	0.78–1.97	0.360
5	1.410	0.85–2.34	0.180
≥6	2.710	1.83–4.01	<0.001
Hypertension			
Yes	0.950	0.73–1.23	0.680
No	ref		
Diabetes			
Yes	0.890	0.67–1.19	0.440
No	ref		
Heart diseases			
Yes	1.060	0.84–1.34	0.630
No	ref		
Stroke			
Yes	1.610	1.26–2.05	<0.001
No	ref		
Chronic respiratory disease			
Yes	1.330	1.03–1.72	0.030
No	ref		
Cancer			
Yes	1.590	0.89–2.85	0.140
No	ref		
Parkinson’s disease			
Yes	2.450	1.61–3.74	<0.001
No	ref		
Dementia			
Yes	2.740	1.86–4.04	<0.001
No	ref		
Anxiety (GAD-7)			
Yes	3.330	2.6–4.25	<0.001
No	ref		
Depression (GDS-15)			
Yes	3.340	2.63–4.25	<0.001
No	ref		

Comorbidities with psychological disorders had a negative impact on the MTBQ score. There were 528 (33.12%) and 568 (35.63%) individuals prone to suffering from depression and anxiety, respectively (Table 1). High multimorbidity treatment burden was correlated with depression (OR = 3.33, 95% CI = 2.60–4.25) and anxiety (OR = 3.34, 95% CI = 2.63–4.25) (Table 2).

### Correlation between MTBQ score and chronic disease self-efficacy

In total, 510 (31.89%) individuals reported low chronic disease self-efficacy (Table 1). The mean scores of each item of the MTBQ and SEMCD6 are shown in Supplementary Figures 1, 2. The results of the logistic regression are presented in Table 3. After controlling for demographic variables, higher multimorbidity treatment burdens were significantly associated with lower chronic disease self-efficacy [medium burden vs. no burden, OR = 2.93, 95% CI = 2.03–4.24; high burden vs. no burden, OR = 3.73, 95% CI = 2.52–5.52].

**Table 3:** Correlation between MTB and chronic disease self-efficacy.

Level of MTBQ	Model1			Model2		
	OR^1^	95%CI	*p*	OR^2^	95%CI	*p*
Non	ref			ref		
Low	1.87	1.45–2.42	<0.001	1.74	1.34–2.26	<0.001
Medium	2.93	2.03–4.24	<0.001	2.36	1.62–3.45	<0.001
High	3.73	2.52–5.52	<0.001	2.63	1.74–3.97	<0.001

^1Odds ratio adjusted for gender, age, residence, high school educational level.^

^2Odds ratio adjusted for gender, age, residence, high school educational level, anxiety, and depression.^

Considering the impact of psychological factors (anxiety and depression) on health behaviors, anxiety and depression were included as control variables in the logistic regression analysis. After controlling for these psychological factors, the correlation between each level of MTBQ score and chronic disease self-efficacy remained significant, with a slight decrease in the correlation coefficient. This indicates that the effect of multimorbidity treatment burden on chronic disease self-efficacy may be partly influenced by mental factors. The result of the correlation between psychological factors and chronic disease self-efficacy is illustrated in Supplementary Table 2.

## Discussion

This study aims to investigate the treatment burden of multimorbidity among elderly individuals from the perspectives of medication and treatment, medical-related, and daily self-health management, identify factors influencing this burden, and explore its relationship with chronic disease self-efficacy. In this study, a patient-centered tool was employed to assess treatment burden in elderly individuals with multimorbidity. The 12-item Chinese version of the Multimorbidity Treatment Burden Questionnaire (MTBQ) was convenient for older individuals to complete independently (25, 35). To the best of our knowledge, this is the largest sample of the MTBQ among the Chinese elderly. Particularly for those aged 80 years and above, the proportion in this age group was 13.01% (208/1,599), and the maximum age of the sample was 95 years (average age: 71 years old).

The possibility of chronic comorbidity was increased with age, particularly among individuals lacking appropriate and timely healthcare (36). Treatment burden varied by gender, indicating that women often bear a greater burden due to their roles in housekeeping and caregiving (37, 38). Higher education levels were associated with a higher socioeconomic status, which may reduce the economic burden of treatment, promote the maintenance of healthy habits and self-medication practices, and provide better access to healthcare services (39–41). Additionally, rural residents were more susceptible to a higher multimorbidity burden than their urban counterparts due to lower income and poor medical resources (42).

In our study, we found that a higher number of chronic diseases was associated with increased treatment burden, which was consistent with previous studies (9, 24, 43). Treatment burden was positively associated with stroke and pulmonary disease (bronchitis, emphysema, pneumonia, and asthma), which was possibly due to individuals perceiving difficulties in managing healthcare tasks (44, 45). This finding differed from a previous MTBQ study, possibly because our study included a large number of older individuals (9). Moreover, patients with Parkinson’s disease and dementia were more likely to report higher MTBQ scores, potentially due to impaired cognitive function and increased healthcare needs (44).

Comorbidities with mental disorders are common in the elderly population and are widely recognized in clinical guidelines for managing multimorbidity (46, 47). We found that comorbidities with anxiety or depression increased the risk of patients experiencing a higher treatment burden. Depression led to a decline in patients’ subjective health, particularly when comorbid with multiple non-communicable diseases (NCDs). This may be attributed to depression amplifying the risk factors of disease and having an additive effect on disease burden (8, 48, 49). Furthermore, psychological disorders also impair patients’ motivation for healthcare and health management, and this effect may be exacerbated in later life due to the increased use of multiple medications (37, 50–52).

The Self-Efficacy for Managing Chronic Disease 6-Item Scale (SEMCD6) was employed to assess the self-efficacy of participants in managing chronic conditions. This instrument has been validated across diverse chronic diseases and enables the precise identification of specific difficulties and needs encountered by patients during symptom management and general disease management (53). Our observations revealed that a higher MTBQ score was associated with lower chronic disease self-efficacy. The findings are consistent with previous studies, indicating that a higher level of self-management efficacy in chronic diseases positively correlates with better symptom management and positive health behaviors (54–56). Moreover, psychological distress and inadequate social support were negatively correlated with a lower level of self-management efficacy in chronic diseases (57, 58). The reason may be that a higher treatment burden impacts people’s confidence in taking steps to overcome the adverse effects of chronic diseases (16, 50, 59). The financial and caregiving burden of multimorbidity treatment may leave patients with a sense of relative deprivation, thereby reducing their confidence and expectations (60).

Moreover, after controlling for psychological variables, including anxiety and depression, the correlation between MTBQ score and low level of chronic disease self-efficacy remained significant, with a slight decrease in the correlation coefficient. Self-efficacy is related to patients’ ability to utilize relevant resources to complete tasks and is based on confidence and behavioral expectations (5, 11). It is closely related to self-management behaviors, including self-care actions, regular medication, and utilization of medical services (61–63). Depression and anxiety may cause individuals to intentionally disobey treatment tasks, increasing the difficulty of disease management (64).

## Conclusion

This study examined the risk factors associated with treatment burden among elderly individuals, considering socioeconomic factors and disease conditions. Individuals with lower socioeconomic status and comorbidities such as stroke, pulmonary disease, cognitive diseases, and psychological disorders may perceive a higher treatment burden of multimorbidity, which could consequently lead to poor health management behaviors. Furthermore, we tentatively conclude that psychological factors play a role in the impact of treatment burden on chronic disease self-efficacy.

## Limitations

To the best of our knowledge, this study represents the largest population-based investigation employing the Multimorbidity Treatment Burden Questionnaire (MTBQ) among the Chinese elderly. At the same time, several limitations should be acknowledged. First, the self-reported data collected through investigation may introduce recall bias, particularly among older participants. Second, elderly respondents may face challenges understanding certain questions due to cognitive decline or other age-related factors, potentially leading to response biases. Finally, information about the year of chronic disease multimorbidity was not investigated, and the discrepancies in results across various disease patterns warrant further investigation.

Future research should aim to incorporate a broader range of variables closely associated with the treatment burden of multiple chronic diseases, such as healthcare services, social support networks, and lifestyle factors, to provide a more comprehensive understanding of the mechanisms underlying chronic disease burden. Additionally, employing mixed-methods approaches, including qualitative interviews and longitudinal designs, could help mitigate recall bias and capture the dynamic nature of chronic disease progression. By addressing these gaps, future studies can offer deeper insights into the multifaceted effects of multimorbidity treatment burden of chronic diseases and inform more targeted interventions to improve the health and wellbeing of aging populations.

## Data Availability

The raw data supporting the conclusions of this article will be made available by the authors, without undue reservation.
