# Supplementary material for: Relationship between multimorbidity treatment burden and chronic disease self-efficacy in the elderly population of China
Source: Front Med (Lausanne). 2025 Apr 3;12:1521189. doi: 10.3389/fmed.2025.1521189 (PMC12004411; doi:10.3389/fmed.2025.1521189)
Supplement: Supplementary file 1 [file Data_Sheet_1.docx]

**Supplementary Appendix**

**Table1** Responses of the questionnaire about multimorbidity treatment burden

| Please tell us how much difficulty you have with the following: | N | Not difficult n (n/N%) | A little difficult  n (n/N %) | Quite difficult  n (n/N %) | Very difficult  n (n/N %) | Extremely difficult  n (n/N %) | Does not apply n (n/N %) |
| --- | --- | --- | --- | --- | --- | --- | --- |
| 1. Taking lots of medications | 1599 | 1254(78.42) | 148(9.26) | 76(4.75) | 40(2.5) | 33(2.06) | 48(3) |
| 2. Remembering how and when to take  medication | 1599 | 1378(86.18) | 124(7.75) | 40(2.5) | 21(1.31) | 18(1.13) | 18(1.13) |
| 3. Paying for prescriptions, over the counter  medication or equipment | 1594 | 997(62.55) | 250(15.68) | 100(6.27) | 55(3.45) | 56(3.51) | 136(8.53) |
| 4. Collecting prescription medication | 1593 | 1134(71.19) | 166(10.42) | 67(4.21) | 46(2.89) | 36(2.26) | 144(9.04) |
| 5. Monitoring your medical conditions | 1588 | 1071(67.44) | 210(13.22) | 82(5.16) | 56(3.53) | 46(2.9) | 123(7.75) |
| 6. Arranging appointments with health professionals | 1589 | 1233(77.6) | 168(10.57) | 76(4.78) | 43(2.71) | 32(2.01) | 37(2.33) |
| 7. Seeing lots of different health professionals | 1593 | 1110(69.68) | 188(11.8) | 81(5.08) | 67(4.21) | 45(2.82) | 102(6.4) |
| 8. Attending appointments with health  professionals | 1592 | 1099(69.03) | 216(13.57) | 65(4.08) | 54(3.39) | 52(3.27) | 106(6.66) |
| 9. Getting help from community services | 1592 | 1070(67.21) | 169(10.62) | 77(4.84) | 46(2.89) | 54(3.39) | 176(11.06) |
| 10. Obtaining clear and up-to-date information  about your condition | 1594 | 1171(73.46) | 175(10.98) | 84(5.27) | 51(3.2) | 34(2.13) | 79(4.96) |
| 11. Making recommended lifestyle changes | 1594 | 1251(78.48) | 180(11.29) | 52(3.26) | 30(1.88) | 23(1.44) | 58(3.64) |
| 12. Having to rely on help from family and  friends | 1597 | 1209(75.7) | 149(9.33) | 44(2.76) | 25(1.57) | 29(1.82) | 141(8.83) |

**Table2** Correlation between psychological factors and chronic disease self-efficacy

| **psychological factors** |  | **OR^1^** | **95%CI** | **p** |
| --- | --- | --- | --- | --- |
| anxiety | no | ref |  |  |
|  | yes | 1.61 | 1.21-2.15 | 0.001 |
| depression | no | ref |  |  |
|  | yes | 2.41 | 1.77-3.28 | <0.001 |

Note:

1. Odds ratio adjusted for gender, age, residence, high school educational level.

**Figure 1** Mean score of each MTBQ item


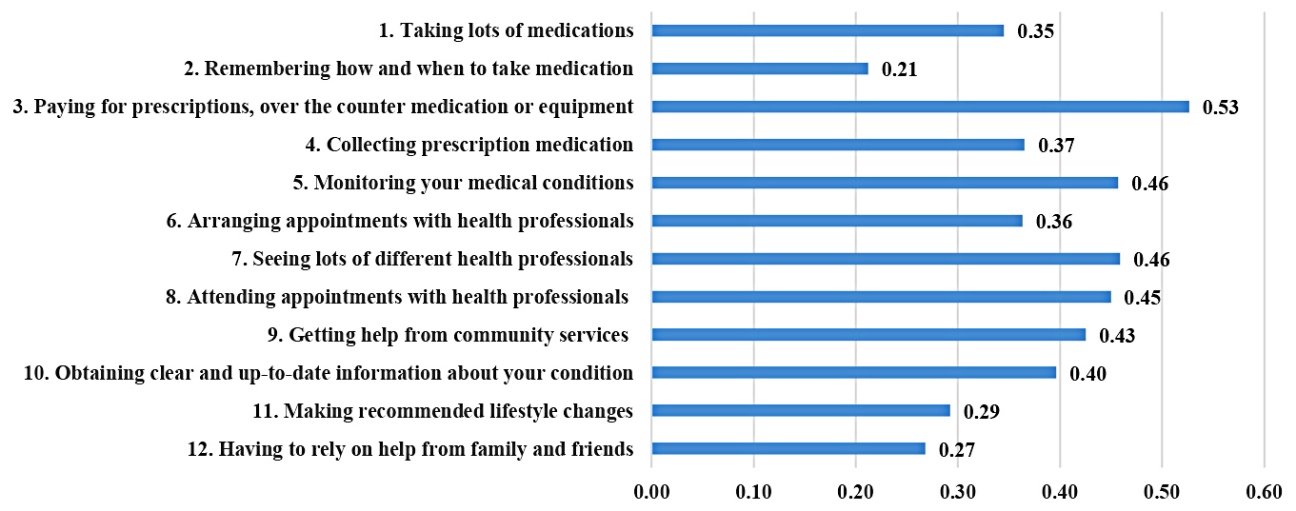


Note: Each item of the MTBQ ranged from 0 (not difficult or not apply)-4 (extremely difficult).

**Figure 2** Mean score of each SEMCD6 item


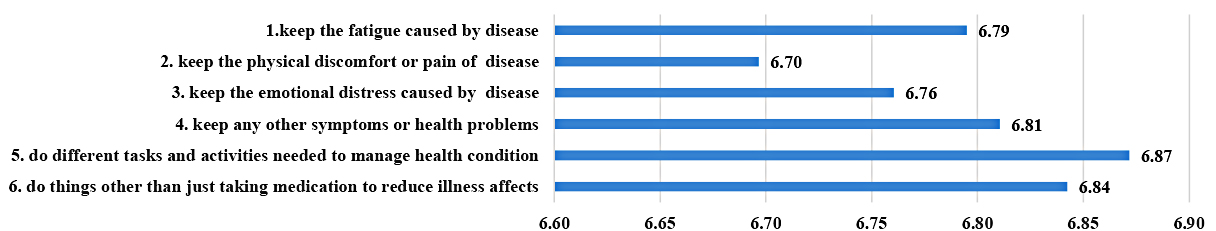


Note: Each item of the SEMCD6 ranged from 1 (not at all confident)-10(totally confident).
